# Reviewed article: Alencar JVA, Carvalho DPC, Umeno HG, França MASA, Azevedo MN. Intersectional analysis of the social vulnerability of individuals with differences in the time between oral cancer diagnosis and treatment: a cross-sectional study, Brazil, 2011-2020. Epidemiol Serv Saude. 2025:34;e20240849

**DOI:** 10.1590/S2237-96222025v34e20240849.b

**Published:** 2025-09-01

**Authors:** Arn Migowski

**Affiliations:** 1 Instituto Nacional de Câncer, Rio de Janeiro, RJ, Brazil

## Peer review B

## First round comments

O artigo trata de um tema relevante para a saúde pública.

Os resultados giram em torno dos seguintes perfis, abarcando quase todas as análises:

perfil (A) de homens predominantemente brancos (96,9%) com escolaridade menor que 8 anos (59,7%), da região Sul (51,5%), casados ou em união consensual (61,4%), com com idade entre 40 a 79 anos;

um perfil (B) de homens negros (96,7%), com escolaridade de 8 anos (53,6%), da região Nordeste (43,3%), casados ou em união consensual (50,2%) e com idade entre 40 a 79 anos

perfil (C) de homens negros (99,7%), com escolaridade menor que 8 anos (100%), da região Nordeste (48,6%),casados ou em união consensual (55,4%), com idade entre 40 a 79 anos; e por fim, um perfil

perfil (D) de mulheres negras (59,3%), com escolaridade menor que 8 anos (85,1%), da região Sudeste (35,5%), viúvas (84,3%) e com idade entre 40 a 79 anos

A discussão sobre os resultados torna-se difícil diante desses perfis. Tanto que a discussão acaba sendo mais tradicional em cima de cada variável sem tanto respaldo dos resultados. Recomendo explorar melhor possíveis explicações para os resultados encontrados para os perfis. Recomendo também que haja um modelo tradicional com análise multivariada controlando o confundimento das diversas variáveis, seja para substituir uma das tabelas com perfis, seja ao menos como material suplementar. Assim seria possível explorar se cada uma dessas variáveis tem ou não ao menos um papel de preditor independente para o desfecho. Da forma que os resultados foram apresentados isso não fica claro.

Recommendation: Major revision

